# Planetary health literacy as an educational goal contributing to healthy living on a healthy planet

**DOI:** 10.3389/fmed.2024.1464878

**Published:** 2024-08-29

**Authors:** Carmen Jochem, Julia von Sommoggy, Anna-Katharina Hornidge, Eva-Maria Schwienhorst-Stich, Christian Apfelbacher

**Affiliations:** ^1^Department of Planetary & Public Health, Faculty of Law, Business & Economics, University of Bayreuth, Bayreuth, Germany; ^2^Institute of Social Medicine and Health Systems Research, Otto-von-Guericke University Magdeburg, Magdeburg, Germany; ^3^German Institute of Development and Sustainability, Bonn, Germany; ^4^Department for Political Sciences and Sociology, University of Bonn, Bonn, Germany; ^5^Department of General Practice, Working Group Climate and Planetary Health, University Hospital Würzburg, Würzburg, Germany; ^6^Nanyang Technological University Singapore, Lee Kong Chian School of Medicine Clinical Sciences Building, Singapore, Singapore

**Keywords:** planetary health literacy, education, planetary health, sustainable development, health professionals

## 1 Introduction

Education plays a key role for sustainability, and thus for healthy living on a healthy planet (1, 2). In particular, education for sustainable development (ESD) and planetary health education (PHE) can unfold their transformative potential for sustainable planetary health through the provision of information and passing on of knowledge, attitudes and skills relating to the interconnection between sustainable development, environment and human health (1). Within ESD and PHE, enabling individuals to develop competencies and practical skillsets to make decision that are environmentally sensitive and health promoting could be a key educational goal.

## 2 Toward planetary health literacy

However, existing literacy concepts that aim to improve such competencies mostly remain within their inherent perspectives and disciplines. Whereas health literacy exclusively refers to human health, environmental literacy refers to wellbeing in a broader context than specifically to individual human health (3, 4). In ESD and PHE, sustainability and transformative literacy are the concepts predominantly used. Although these concepts include crucial aspects for sustainable development and transformative change, they do not explicitly address environmental and human health aspects. Thus, the strong interconnection between human health and the environment—in the sense of planetary health—is not reflected adequately by existing literacy concepts.

Considering the lack of a comprehensive and integrative literacy concept that includes all relevant aspects, we proposed a conceptual model of planetary health literacy (PHL) (5), defining PHL as “the knowledge and competencies of accessing, understanding, appraising, and applying information in order to make judgements and take decisions regarding planetary health, across societies and for health-promoting, sustainable, and transformative actions. Planetary health literate individuals and societies are enabled to sustain and promote their own health, population health, and the planet's health. They are able to adopt a more holistic understanding of their health embedded in natural systems they are living in. Based on their knowledge and attitude, they take decisions that reflect and foster the interconnectedness of human health and wellbeing with the state of the natural systems and related areas of nature-society interactions” (5). By encompassing both a life-course and transgenerational approach, PHL may act across multiple current and future generations. Thereby, PHL goes beyond the individual level and includes a societal and global level.

## 3 Planetary health literacy as an educational goal

**Figure 1:**
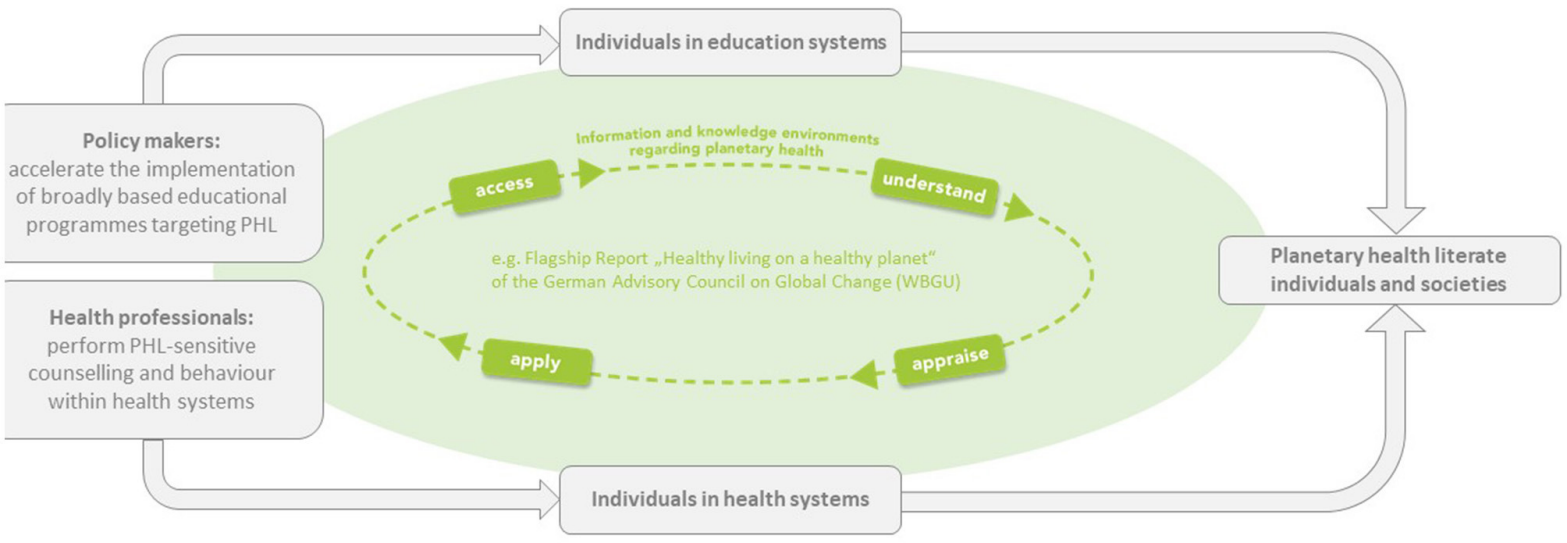
Role of PHL for the vision of healthy living on a healthy planet. Policy makers and health professionals (among others) can serve as relevant multipliers of PHL within the education and health system. Their knowledge and competencies enable them to behave in a planetary health literate way, i.e., to access, understand, appraise, and apply information and knowledge environments regarding planetary health—such as the recommendations for “Healthy living on a healthy planet” by the German Advisory Council on Global Change. Implementing these recommendations would contribute to learning and knowledge environments that help individuals—within the education system and the health system—to develop PHL and to make decisions for health promoting and sustainable lifestyles.

Education fostering PHL could enable individuals and societies to positively contribute to the vision of healthy living on a healthy planet and to the associated transformation to sustainability (1). To achieve this vision, the German Advisory Council on Global Change—an independent scientific advisory body of the German government—recommends political actors to systematically promote education for healthy living on a healthy planet worldwide (1). Using PHL as goal in an education strategy should “enable and promote knowledge, attitudes and skills relating to environmental and human health throughout life, and […] encourage sustainable action within the educational institutions themselves” (1). The existing processes of embedding ESD in all areas of education—from pre-school to advanced-training programmes—and of implementing PHE not only in health-related educational programmes, should be reinforced and policy makers should accelerate the implementation of new broadly based educational strategies targeting PHL as soon as possible (1). Health professionals would contribute to environmentally sensitive health promotion and prevention through PHL-sensitive counseling [e.g., climate-sensitive health counseling (6)] and to sustainable health systems through sustainable behavior and use of resources. Within these information and knowledge environments, individuals, and societies could become planetary health literate and, for example, make more health promoting and sustainable decisions regarding nutrition or mobility. Figure 1 illustrates the role of PHL for the vision of healthy living on a healthy planet.

## 4 Discussion and research needs

Such a vision does not only require political and societal action, but is accompanied by several research needs: First, PHL itself needs to be investigated in more detail. The conceptual model needs to be empirically validated and a set of indicators need to be determined to assess PHL. Second, inter- and transdisciplinary research projects that investigate in a participatory manner how educational programmes can be developed and implemented to enable PHL across the life-course are needed [e.g., in real-world laboratories (7)]. Third, the implications of different ways of knowing, power dynamics, justice and equity on planetary health literacy need to be understood.

In conclusion, the prompt implementation of recommendations—not only related to education—of scientific advisory bodies such as the global urgency governance emphasized by the German Advisory Council on Global Change are crucial for healthy living on a healthy planet. The concept of planetary health literacy can serve as an educational goal contributing to this vision.

## References

[1] German Advisory Council on Global Change. Healthy Living on a Healthy Planet. Berlin: WBGU (2023).

[2] United Nations. Transforming our world: The 2030 Agenda for Sustainable Development. New York, USA: UN (2015).

[3] Sørensen K Van den Broucke S Fullam J Doyle G Pelikan J Slonska Z. Health literacy and public health: a systematic review and integration of definitions and models. BMC Public Health. (2012) 12:80. 10.1186/1471-2458-12-80 22276600 PMC3292515

[4] Hollweg KS TJ BR Marcinkowski TJ McBeth WC Zoido P. Developing a Framework for Assessing Environmental Literacy. Washington, DC: North American Association for Environmental Education. (2011).

[5] Jochem C von Sommoggy J Hornidge AK Schwienhorst-Stich EM Apfelbacher C. Planetary health literacy: a conceptual model. Front Public Health. (2022) 10:980779. 10.3389/fpubh.2022.980779 36726624 PMC9886088

[6] Quitmann C Griesel S Nayna Schwerdtle P Danquah I Herrmann A. Climate-sensitive health counselling: a scoping review and conceptual framework. Lancet Planet Health. (2023) 7:e600–e10. 10.1016/S2542-5196(23)00107-9 37438001

[7] Wanner M Hilger A Westerkowski J Rose M Stelzer F Schäpke N. Towards a cyclical concept of real-world laboratories. disP Plan Rev. (2018) 54:94–114. 10.1080/02513625.2018.1487651

